# Combination of waveforms in modern spinal cord stimulation

**DOI:** 10.1007/s00701-021-05107-4

**Published:** 2022-01-05

**Authors:** Piedade G. S, Gillner S., Slotty P. J., Vesper J

**Affiliations:** 1 Department of Neurosurgery, Heinrich-Heine-Universität Düsseldorf, Moorenstr. 5, 40225 Düsseldorf, Germany; 2 Department of Functional Neurosurgery and Stereotaxy, Heinrich-Heine-Universität Düsseldorf, Düsseldorf, Germany

**Keywords:** Spinal cord stimulation, Waveform, Combination therapy, Neuropathic pain

## Abstract

**Background:**

After the surge of burst stimulation, different waveforms were developed to optimize results in spinal cord stimulation. Studies have shown higher responder rates for multiwave therapy, but since the launch of such multiwave systems, little is known about the patients’ preference regarding waveforms in the long-term follow-up. No study connected particular waveforms to specific pain etiologies or required stimulation parameters so far.

**Method:**

Thirty-four patients with refractory chronic neuropathic pain were treated with spinal cord stimulation systems providing multiwave therapy between September 2018 and October 2019. Patients with a follow-up of at least 6 months were selected; 10 subjects were excluded due to revision surgery, infection, and loss to follow-up. Data regarding pain intensity and preferred waveform for the trial, the implantation, 3-month and 6-month follow-up were recorded.

**Results:**

During the trial phase, 10 patients (43.5%) achieved significant pain relief using tonic stimulation, 5 using burst (21.7%), 3 using microburst (13.0%), and 4 using a combination of tonic and microburst (17.4%). One single patient preferred Contour stimulation during the trial. After 3 months, 6 patients preferred microburst (25%), 6 preferred tonic (25%), 5 used a combination of tonic and microburst (20.8%), and 5 patients used burst (20.8%). After 6 months, similar results were obtained. Contour and Whisper were used in complex cases failing to other waveforms.

**Conclusions:**

Tonic stimulation, isolated or in combination, remains an important component in spinal cord stimulation, being used by almost half of the patients. Over time, the usage of microburst increased considerably. Whisper and Contour, although battery-consuming, are good salvage options in complex cases.

## Introduction

The field of spinal cord stimulation (SCS) developed in recent years with new waveforms targeting different needs in the therapy of neuropathic pain. Tonic stimulation (10–150 Hz) was the very first to be developed and provides mostly paresthesia in the stimulated area, which is very useful when evaluating the coverage of the painful area during the first programming session. Burst stimulation was later developed and its first form consisted of five stimulus bursts, classically with an intraburst frequency of 500 Hz, delivered with a frequency of 40 Hz under constant amplitude [3]. It provided significant pain relief to patients previously using classic tonic stimulation [2]. After the surge of burst stimulation that made a paresthesia-free stimulation possible, alternatives such as microburst, Whisper, and Contour appeared and gained relevance in the neuromodulation of the spinal cord (Table 1) [1]. Commercially available systems were developed to provide multiwave therapy—some of them even simultaneously [6]. Systems that allow patients to choose among multiple waveforms were reported to have high responder rates even in previously implanted patients that failed to trials with high-dose SCS [4]. Since the launch of such multiwave systems, little is known about the patients’ preference regarding waveforms in the long-term follow-up. We aim to report our experience with the programming of spinal cord stimulators in the context of multiwave therapy, identify the prevalence of different waveforms over time, and critically discuss the importance of each of them in the treatment of chronic pain.

**Table 1:** Waveforms provided by the tested devices

	Definition
Tonic	Regular stimuli generally applied with a frequency of 10 to 150 Hz, amplitude up to 25 Hz
Burst	Intermittent packets of burst stimuli with 2–7 pulses per packet, interburst frequency of 40 Hz and pulse widths up to 1000 μs, amplitude at 60% of the perception threshold
Microburst	Customized stimulation with 2–1000 pulses per packet, 1–80 Hz interburst frequency, and up to 1200 Hz intraburst frequency
Contour	Algorithm that shapes the stimulation field over multiple vertebral levels to the spinal anatomy and lead position
Whisper	High-frequency tonic stimulation between 1000 and 1200 Hz, amplitude at 60% of the perception threshold

## Methods and materials

Patients with chronic neuropathic pain that were treated with spinal cord stimulation systems providing multiwave therapy between September 2018 and October 2019 in our center were preselected; those achieving at least 6 months of follow-up were included in the study. Data regarding pain intensity and waveform in use for the trial, the implantation, and the follow-up appointments were collected. Pain intensity was assessed with the visual analog scale (VAS).

Implanted systems were Spectra Wavewriter™ and Precision Montage™, both manufactured by Boston Scientific Corporation (Marlborough, MA, USA). The first system was used in 13 patients and is the only one to allow simultaneous treatment with two different waveforms using different electrodes. In both systems, the percentage of electric current running through each individual electrode can be controlled.

This study was approved by the local IRB (2020–905). We used descriptive statistics in this study.

## Results

A total of 34 patients were treated with a spinal cord stimulation system providing multiwave therapy; 10 subjects however did not achieve 6 months of follow-up due to revision surgery, infection, and loss to follow-up and were therefore excluded from the analysis. We analyzed a study pool of 24 patients. Twenty-three patients were submitted to a trial of spinal cord stimulation; in one case, an older IPG was substituted by a device with multiwave therapy. A trial with temporary lead in an out-patient setting was done in 14 patients; in these cases, the trial lasted for 14 days. The remaining 9 patients were submitted to a trial with permanent lead; the trial phase lasted for a maximum of 14 days but could be interrupted earlier for implantation of the IPG. Test of each waveform during the trial lasted for approximately 4 days each. Patients under multiwave therapy had a mean age of 61.8 years at the implantation of the pulse generator (IPG) and were predominantly female (66.7%); mean follow-up of 6.8 months was seen (Table 2). The time period between trial and implant was on average 19.3 days. Failed back surgery syndrome responded for 15 of the indications for spinal cord stimulation (62.5%); mean pain intensity at the baseline was 8.1. A successful trial stimulation was obtained in 10 patients with tonic stimulation (43.5%), followed by burst (5, 21.7%), tonic and microburst (4, 17.3%), microburst (3, 13.0%), and Contour (1, 4.3%). Patient 13 was already under SCS with another system; in a revision surgery, the system was changed without a previous trial using multiwave therapy. After implantation of the IPG, patients were discharged with similar stimulation parameters. Tonic stimulation was rejected by three patients and a good response was seen in 7 cases (29.2%); microburst was chosen by two more individuals and was chosen by 5 discharged patients (20.1%). One single patient was discharged alternating frequently between tonic and burst; the IPG in this case could not provide simultaneous stimulation with both waveforms.

**Table 2:** Pain intensity and preferred waveform over time

	Age	Sex	Pain etiology	Lead level	Baseline		Implantation Waveform	3 months f/u		6 months f/u		12 months f/u	
					VAS	Waveform		VAS	Waveform	VAS	Waveform	VAS	Waveform
1	51	F	Low back pain	T9	7	Tonic	Tonic	3	MB	0	Tonic/MB		
2	52	M	FBSS	T9 and T7	9	Tonic/MB	Tonic/MB	7	Tonic/MB	3	Tonic/MB	2	Tonic/MB
3	71	M	Post-myelitis	C5	8	Tonic/MB	Tonic/MB	5	Tonic/MB	3	Whisper		
4	58	M	FBSS	T9	8	MB	MB	3	MB	3	Tonic/MB		
5	81	F	FBSS	T8	8	MB	MB	8	Tonic/MB	4	Contour		
6	78	F	FBSS	T9	8	Contour	Contour	8	MB	8	MB		
7	77	F	Sciatica	T9	8	Tonic/MB	MB	2	MB	4	MB		
8	84	F	Sciatica	T9	9	Tonic	Tonic	5	Tonic	4	Tonic		
9	70	F	FBSS	T8	8	Burst	Tonic/MB	4	Tonic/MB	8	Tonic/MB		
10	67	F	FBSS	T8	8	Tonic	MB	1	MB	1	Tonic/MB		
11	70	F	FBSS	T7	9	MB	MB	3	Burst/MB	5	Burst/MB		
12	71	F	FBSS	T8	9	Tonic/MB	Tonic/MB	2	Tonic/MB	2	MB		
13	80	F	FBSS	T9	8	-	MB	9	MB	8	MB		
14	59	M	Sciatica	T9	7	Tonic	Tonic	3	Tonic	7	Tonic		
15	48	F	Sciatica	T9	6	Burst	Burst	4	Burst	6	Burst	5	Burst
16	58	M	FBSS	T8	8	Tonic	Tonic	2	Tonic	4	Burst		
17	47	M	Peripheral neuropathy	T10	9	Burst	Burst	3	Tonic	3	Tonic		
18	51	M	Post-traumatic	T8	9	Burst	Burst	2	Burst	2	Burst		
19	39	F	FBSS	T8	9	Tonic	Burst	0	Burst	7	Tonic/burst		
20	61	F	FBSS	T7	8	Tonic	Tonic	3	Tonic	3	Burst		
21	41	F	FBSS	C4	6	Tonic	Tonic/burst	4	Tonic/burst	2	Tonic		
22	57	M	FBSS	T8	8	Tonic	Tonic	4	Tonic	0	Tonic		
23	59	F	FBSS	C5	7	Tonic	Tonic	6	Burst	4	Whisper		
24	53	F	Sciatica	T8	10	Burst	Burst	3	Burst	3	Burst	6	Burst

Patients 1–13 were implanted with Spectra WaveWriter™ and could choose two different waveforms to act simultaneously; patients 14–24 were implanted with Precision Montage™. Lead level indicates the vertebral level of the tip of the lead. Subjects are presented in aleatory order. *FBSS* failed back surgery syndrome, *MB* microburst

After 3 months of follow-up, mean VAS decreased to 3.9, a mean reduction of 51.9% in pain intensity. Overall, 15 patients (62.5%) had a significant pain relief of at least 50%. Tonic and microburst were the most used waveforms, each preferred by 6 patients (25%). Burst and the combination of tonic and microburst were used by 5 patients each (20.8%); the combinations of tonic and burst and of burst and microburst were the case of one patient each (4.2%).

At 6 months follow-up, mean VAS stabilized at 3.9 and 16 patients (66.7%) achieved pain relief of at least 50%. Tonic stimulation, burst, and the combination of tonic and microburst were preferred by 5 patients each (20.8%) and were followed by microburst alone (4, 16.7%). Whisper was used by 2 patients (8.3%); Contour and the combinations of burst with microburst and of burst with tonic were preferred by one patient each (4.2%).

Table 3 gives a better overview of the prevalence of different waveforms over time. Isolated tonic stimulation was the most frequent waveform in successful trials, but lost half of its patients for other waveforms. Burst and microburst kept roughly a fifth of the patients during the entire duration of the observation period. When combinations are also considered, however, tonic stimulation was an important component of the therapy in 14 patients (60.8%) and at 6 months follow-up still was used by 11 (45.8%), being the most used waveform in this study. Microburst initially was used by 7 individuals (30.3%), progressed to 12 at 3 months follow-up (50%) and at the end responded for 10 study subjects (41.7%). Burst was rarely used in combination with other waveforms; its prevalence grew up from 5 (21.7%) to 7 subjects (29.2%) at 6 months follow-up.

**Table 3:** Prevalence of waveforms over time

	Trial	Implantation	3 months f/u	6 months f/u
Tonic	43.5%	29.2%	25.0%	20.8%
Burst	21.7%	20.8%	20.8%	20.8%
Microburst	13.0%	20.1%	25.0%	16.7%
Contour	4.3%	4.2%	0%	4.2%
Whisper	0%	0%	0%	8.3%
Tonic + microburst	17.3%	16.7%	20.8%	20.8%
Tonic + burst	0%	4.2%	4.2%	4.2%
Burst + microburst	0%	0%	4.2%	4.2%

## Discussion

This study observed the phenomenon of a decrease in the prevalence of tonic stimulation alone over time, favoring burst, microburst, or a combination. Many patients sense stimulation-induced paresthesia of tonic stimulation as a comfortable feeling. If there is a good coverage and patients do not need higher current intensities, many patients remain using exclusively tonic. In the case, however, that sufficient pain relief can only be achieved using higher current intensities; subperception therapies are generally preferred, such as burst stimulation and microburst. Both waveforms allow a higher tissue activation without the side effects of tonic stimulation using high amplitudes. The same occurs in the case of insufficient coverage of the painful area, when higher amplitudes try to compensate the pain of the uncovered region. That is possibly the reason why combinations between tonic and burst or microburst are frequent during the initial programming and get even more frequent over time.

When considered together, burst stimulation and microburst were used by 12 patients during the trial (52%) and after 6 months were a component of the therapy in 17 of them (70.9%). An alternative to burst that reduces battery consumption is microburst, with an intraburst frequency of 450 Hz. Microdosing burst stimulation with alternation of ON and OFF periods was also a successful strategy [10].

In this study, two patients with cervical leads used Whisper stimulation at a frequency of 1 kHz after tonic and burst stimulation failed to provide sufficient pain relief. One subject achieved significant pain relief; the other one had a reduction of 42.8% in pain intensity. High-frequency spinal cord stimulation was initially tested for 10 kHz with superiority to conventional tonic stimulation in the SENZA trial [5]. Equivalent pain relief, however, was obtained using 1 kHz in the PROCO trial, which reduced battery consumption [9]. The Whisper trial tested frequencies up to 1.2 kHz in previously implanted patients and, when the patients were given the choice, increased pain relief was achieved [7]. High-frequency stimulation has been used particularly in the case of cervical leads with good outcomes [8].

Contour was preferred by only two patients in the study at different times. It is a waveform that shapes the stimulation field to the patients’ anatomy, activating uniformly the dorsal columns despite variabilities in the lead position. Contour is now programmed with 200 Hz and its efficacy has been particularly tested with leads with 1 mm edge-to-edge spacing between electrodes. It is however possible to program it with leads with higher spacing. Contour is a good option in minor lead migration.

This study has the weakness of being retrospective, even considering the high quality of each case’s documentation. If paresthesia coverage and stimulation parameters had been prospectively assessed, more conclusions could have been drawn about the reasons for the use of a specific waveform in each case. This study has however the strength of depicting the preference for waveforms or a combination of them with a high external validity and provides valuable information for the management of stimulation parameters in the context of multiwave therapy in the mid and long term.

## Conclusion

Tonic stimulation, isolated or in combination with other waveforms, is still a very important component of modern SCS. Burst stimulation and microburst represent effective paresthesia-free alternatives. Whisper and Contour, although battery-consuming, are good salvage options in complex cases.

## Abbreviations

SCS: Spinal cord stimulation
VAS: Visual analog scale
IRB: Institutional review board
IPG: Implantable pulse generator
